# Thoracoscopic resection of posterior mediastinal paraganglioma: perioperative management and surgical tips

**DOI:** 10.1186/s13019-022-01892-w

**Published:** 2022-06-06

**Authors:** Yuki Matsumura, Mitsuro Fukuhara, Hayato Tanabe, Hikaru Yamaguchi, Hironori Takagi, Yuki Ozaki, Satoshi Muto, Naoyuki Okabe, Yutaka Shio, Hiroyuki Suzuki

**Affiliations:** 1 Department of Chest Surgery, Fukushima Medical University, 1 Hikarigaoka, Fukushima, 960-1295 Japan; 2 Department of Chest Surgery, Iwaki City Medical Center, Iwaki, Japan; 3 Department of Diabetes, Endocrinology and Metabolism, Fukushima Medical University, Fukushima, Japan

**Keywords:** Posterior mediastinal paraganglioma, Perioperative management

## Abstract

Posterior mediastinal paraganglioma (PM-PGL) is a rare disease that is difficult to diagnose. If PM-PGL is misdiagnosed preoperatively, surgeons may encounter severe tachycardia and hypertension and easy bleeding from the tumor during the operation. Therefore, it is essential to include PGL as a differential diagnosis for mediastinal tumors. We herein describe a 73-year-old Japanese man with a PM-PGL that was diagnosed preoperatively and resected safely by video-assisted thoracic surgery. Preoperative management of hypertension with doxazosin mesylate, soft coagulation of the peritumor area, and careful clipping of feeding arteries were effective for hemostasis. The patient’s vital signs were stable during and after the operation.

**Supplementary Information:**

The online version contains supplementary material available at 10.1186/s13019-022-01892-w.

## Introduction

Posterior mediastinal paraganglioma (PM-PGL) is a rare disease that is difficult to diagnose and manage perioperatively. However, if PM-PGL is preoperatively misdiagnosed, surgeons often encounter severe tachycardia and hypertension and easy bleeding from the tumor during the operation [1]. We herein present a case of PM-PGL that was diagnosed preoperatively and resected safely by video-assisted thoracic surgery (VATS) with stable vital signs and minimal blood loss. We also present surgical tips to consider during this operation.

## Case

**Fig. 1:**
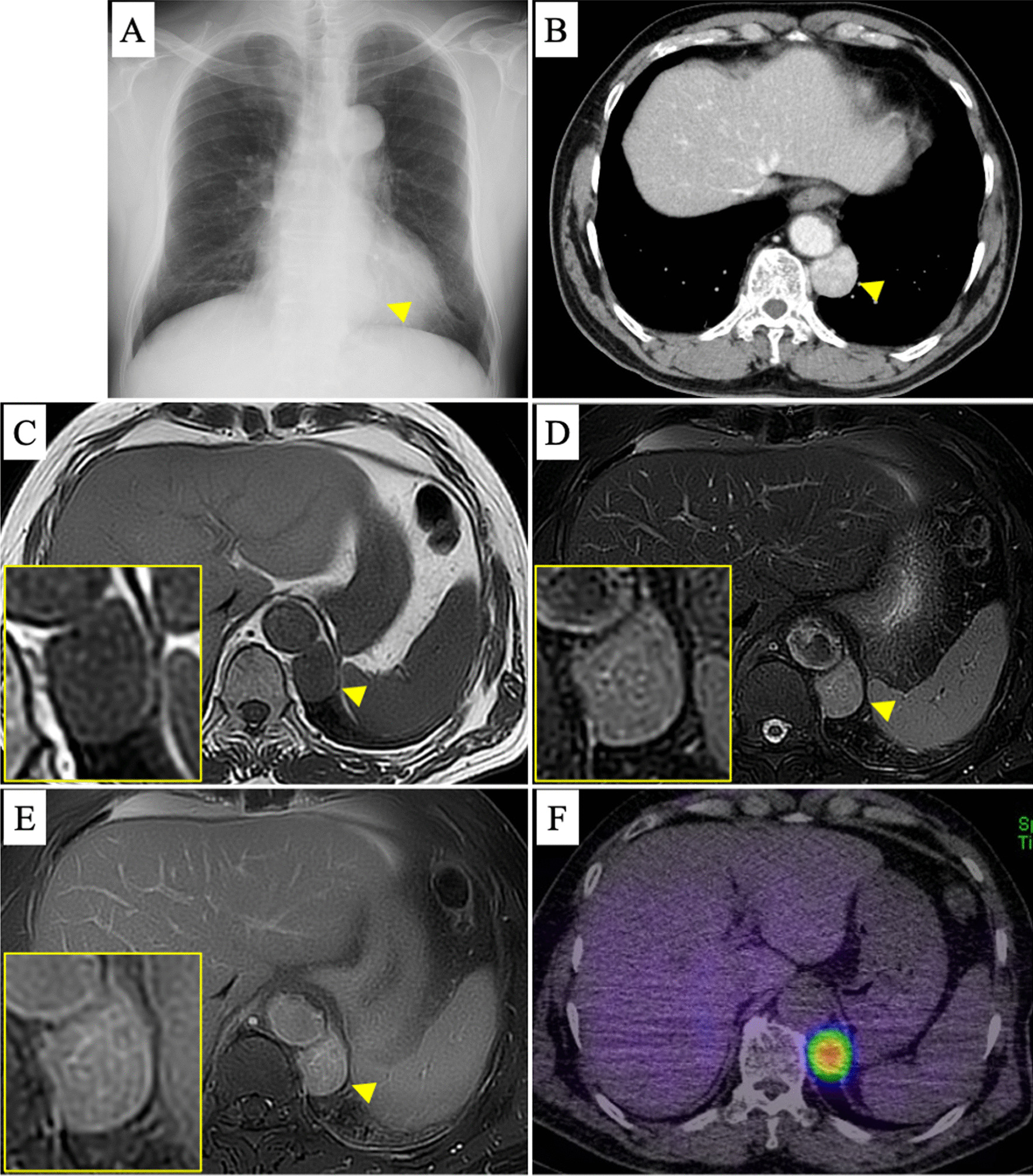
Preoperative radiological images. **A** Plain chest radiograph shows an abnormal mass shadow (arrowhead). **B** CE-CT shows a highly enhanced mass adjacent to the descending aorta (arrowhead). **C**–**E** MRI. The mass shows low intensity on T1-weighted images and high intensity on T2-weighted images with a few flow voids and enhancement by gadolinium. **F** ^131^I-MIBG scintigraphy shows high uptake in the tumor alone

**Fig. 2:**
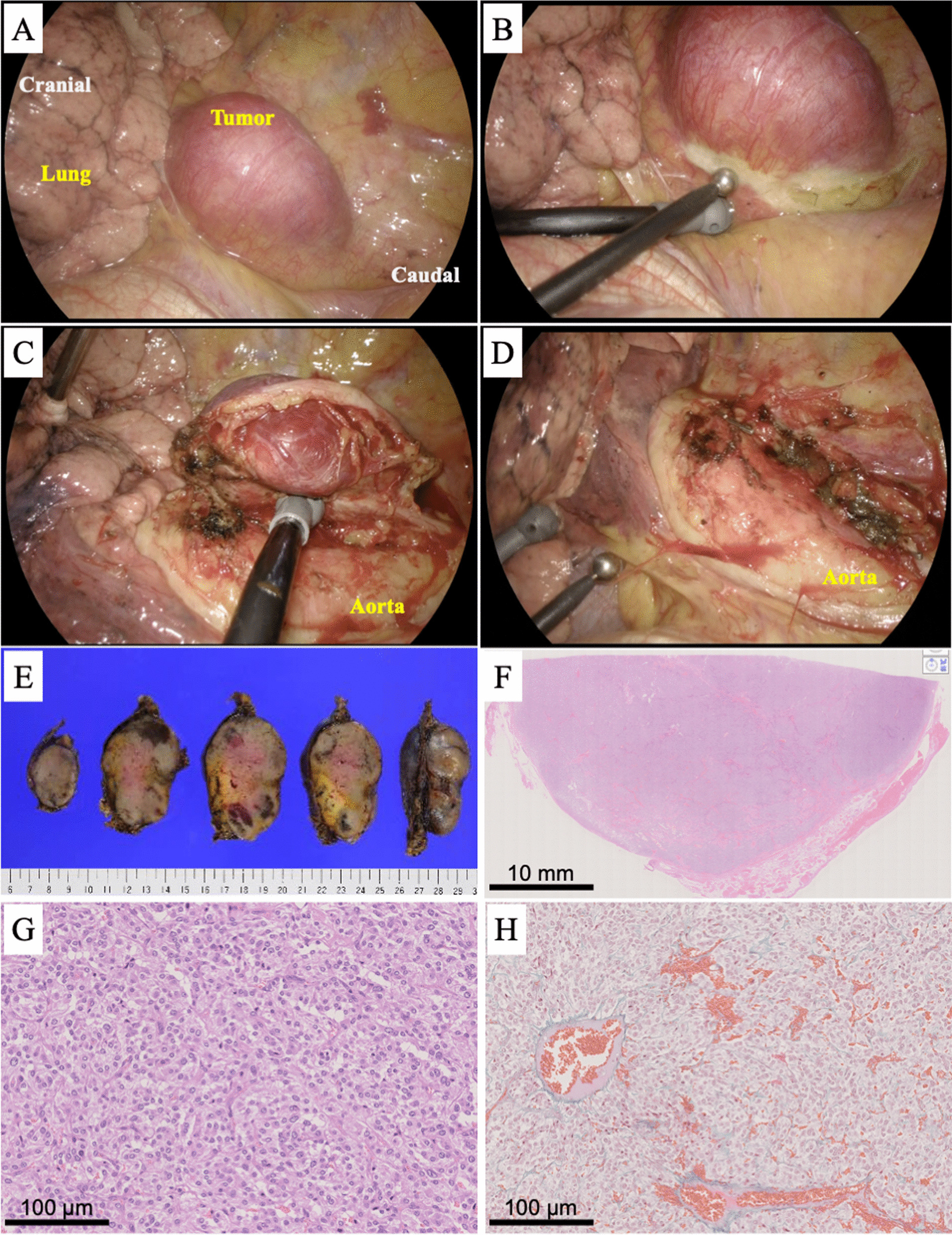
**A**–**D** Intraoperative pictures. Soft coagulation of the peritumoral area and careful clipping of the feeding arteries. **E**–**H** The resected tumor was macroscopically smooth and encapsulated on its surface. Microscopically, the tumor exhibited the so-called Zellballen pattern

A 73-year-old Japanese man visited us because of an abnormal shadow on a plain chest radiograph (Fig. 1A). Contrast-enhanced computed tomography (CE-CT) showed a highly enhanced left posterior mediastinal tumor measuring 3.6 × 2.8 cm (Fig. 1B). On magnetic resonance imaging (MRI), the tumor showed low intensity on T1-weighted images and high on T2-weighted images with a few flow voids and enhancement by gadolinium (Fig. 1C–E). High uptake was observed in the tumor alone on ^131^I-MIBG scintigraphy (Fig. 1F). Hypertension, hyperglycemia, and high urinary normetanephrine (0.61 mg/day) were also observed, although the patient had no PGL-related symptoms such as headache, tachycardia, or nausea. Therefore, the patient was diagnosed with a functional PGL. We performed tumor resection after treating the hypertension for 3 weeks with 4 mg doxazosin mesylate. The VATS approach was started with the patient in the right lateral position. The tumor appeared highly vascular because many small vessels were observed in its capsule. Therefore, soft coagulation of the peritumoral area and careful clipping of the feeding arteries with surgical clips were effective for hemostasis (Fig. 2A–D and Additional file 1). The tumor was safely resected by VATS in 113 min, and the total blood loss was < 10 mL. Vital signs were stable during and after the operation. Wound infection prolonged the hospital stay, but the patient was discharged on postoperative day 31. The urinary normetanephrine concentration decreased to 0.38 mg/day after the operation. Pathologically, the tumor was 4.5 × 3.2 cm in size, and microscopic examination revealed tumor cells with round nuclei and eosinophilic cytoplasm forming nested islands consisting of chief tumor cells, inconspicuous sustentacular cells, and a capillary network (so-called “zellballen” pattern). Therefore, the tumor was diagnosed as PGL, consistent with the preoperative diagnosis (Fig. 2E–H). Although the patient developed no recurrence of the PGL, he was diagnosed with sigmoid colon cancer during postoperative follow-up. He was undergoing therapy for the sigmoid colon cancer at the time of this writing.

## Discussion

The typical clinical features of PGL are hypertension or dizziness due to abnormal production of catecholamines, but PGL is often asymptomatic [2]. In some case reports, the diagnosis was not made until uncontrollable tachycardia, hypertension, and easy hemorrhage occurred during the operation [1, 2]. Therefore, it is essential to include PGL as a differential diagnosis for mediastinal tumors [1, 3]. Once this tumor is suspected, the path to diagnosis is relatively uncomplicated because its characteristic findings can be obtained by CE-CT, MRI, and ^131^I-MIBG scintigraphy [4]. CE-CT is useful for diagnosis because PGL generally appears as a highly enhanced tumor. Some reports suggest that MRI is more useful because flow voids of tumor blood vessels are observed in the relatively higher-signal parenchyma of PGLs, demonstrating the characteristic “salt and pepper” appearance on T1-weighted images [1, 3, 5]. In the current case, a few flow voids were observed in the tumor. For diagnosis, ^131^I-MIBG scintigraphy should be performed and it is useful to confirm multiple pheochromocytomas or PGLs throughout the body. In the present case, the PGL was diagnosed early before the operation because it showed specific radiological findings, which allowed us to prepare for surgical treatment.

Further challenges are the perioperative management of catecholamine-induced tachycardia and hypertension and selection of the surgical approach. Previous articles have indicated that VATS may not be optimal because of the hypervascularity of PGLs [1, 2]. Therefore, we performed strict preoperative medical management of blood pressure, soft coagulation of the peritumoral area, and careful clipping of the feeding arteries with surgical clips. This strategy allowed us to safely resect the tumor by VATS with stable vital signs. Notably, whether the preoperative treatment successfully prevented intraoperative complications such as extremely high blood pressure or severe tachycardia is unclear because the patient’s vital signs were stable perioperatively. However, previous reports of functional PGLs causing intraoperative complications have warned that many such patients were also asymptomatic before the operation [1, 2]. In the current patient, we were at least able to preoperatively suspect a functional PGL, diagnose it radiologically, and safely resect it, which was considered beneficial for the patient.

## Conclusion

We have presented a case in which a PM-PGL was safely resected by VATS. The preoperative diagnosis, perioperative management of hemodynamics, and careful surgical procedures were important for its treatment.

## Supplementary Information

**Additional file 1.** The video shows thoracoscopic resection of PM-PGL. Soft coagulation of the peritumoral area and careful clipping of the feeding arteries with surgical clips were effective for hemostasis.

## Data Availability

Not applicable.
